# Oxidative Stress Biomarkers and Left Ventricular Hypertrophy in Children with Chronic Kidney Disease

**DOI:** 10.1155/2016/7520231

**Published:** 2016-01-18

**Authors:** Dorota Drożdż, Przemko Kwinta, Krystyna Sztefko, Zbigniew Kordon, Tomasz Drożdż, Monika Łątka, Monika Miklaszewska, Katarzyna Zachwieja, Andrzej Rudziński, Jacek Antoni Pietrzyk

**Affiliations:** ^1^Dialysis Unit, Jagiellonian University Medical College, 265 Wielicka Street, 30-663 Krakow, Poland; ^2^Department of Pediatrics, Jagiellonian University Medical College, 265 Wielicka Street, 30-663 Krakow, Poland; ^3^Department of Clinical Biochemistry, Jagiellonian University Medical College, 265 Wielicka Street, 30-663 Krakow, Poland; ^4^Department of Pediatric Cardiology, Jagiellonian University Medical College, 265 Wielicka Street, 30-663 Krakow, Poland; ^5^I Department of Cardiology, Interventional Cardiology and Hypertension, Jagiellonian University Medical College, 17 Kopernika Street, 31-501 Krakow, Poland

## Abstract

Cardiovascular diseases remain the most frequent cause of morbidity and mortality in patients with chronic kidney disease (CKD). The aim of the study was to assess the association between oxidative stress biomarkers and cardiovascular risk factors and left ventricular hypertrophy in children with CKD.* Material and Methods*. The studied group consisted of 65 patients aged 1.4–18.6 (mean 11.2) years with stages 1 to 5 CKD. Serum oxidized low-density lipoprotein (oxLDL), protein carbonyl group, creatinine, cystatin C, albumin, lipids, high-sensitivity C-reactive protein, intercellular adhesion molecule-1, insulin, plasma renin activity, and aldosterone levels were measured. Patients were divided into groups depending on CKD stage. Anthropometric measurements, ambulatory blood pressure (BP) measurements, and echocardiography with left ventricular mass (LVM) calculation were performed.* Results*. Serum oxLDL strongly correlated with creatinine (*R* = 0.246; *p* = 0.048), cystatin C (*R* = 0.346; *p* = 0.006), total cholesterol (*R* = 0.500; *p* < 0.001), triglycerides (*R* = 0.524; *p* < 0.001), low-density lipoprotein concentrations (*R* = 0.456; *p* < 0.001), and 24 hour BP values of systolic (*R* = 0.492; *p* = 0.002), diastolic (*R* = 0.515; *p* < 0.001), and mean arterial pressure (*R* = 0.537; *p* < 0.001). A significant correlation between oxLDL levels and LVM *z*-scores (*R* = 0.299; *p* = 0.016) was found.* Conclusions*. Hypertension and dyslipidemia correlated with lipid oxidation in children with CKD. oxLDLs seem to be valuable markers of oxidative stress in CKD patients, correlating with left ventricular hypertrophy.

## 1. Introduction

An imbalance between the processes of formation of free radicals and their removal with a predominance of the production of reactive oxygen species (ROS) is referred to as oxidative stress. The uncontrolled increase in the concentration of free radicals is postulated to be one of the pathophysiological mechanisms of many diseases such as diabetes, atherosclerosis, vascular dementia, or neoplasms. Under physiological conditions, ROS and reactive nitrogen species (RNS) are constantly produced to defend the body against germs and are also of importance in the processes of cell signaling, tissue healing, and remodeling [1]. ROS include superoxide radical, hydrogen peroxide, and hydroxyl radical. The role of antioxidants in the body is fulfilled by enzymes: superoxide dismutase, catalase, oxidase, and glutathione peroxidase, and other substances such as glutathione, vitamins E and C, magnesium ions, zinc, albumin, ferritin, transferrin, and uric acid.

In patients with chronic kidney disease (CKD) treated conservatively and on dialysis, both the increased production of ROS and RNS and reduced antioxidant status have been shown [2, 3]. Activation of the renin-angiotensin-aldosterone and sympathetic systems, as well as chronic inflammation, results in the production of oxidative stress markers. Low-density lipoprotein particle modified in the process of oxidation (oxLDL) develops atherogenic properties and becomes cytotoxic to vascular endothelial cells, stimulates the growth of smooth muscle, and attracts macrophages. oxLDL also inhibits macrophage mobility favoring their accumulation and formation of fatty streaks—the initial stage of the atherosclerotic process [4–6].

Oxidative stress is considered to be one of the cardiovascular risk factors in patients with CKD. In this group of patients higher prevalence of traditional risk factors (hypertension, dyslipidemia) and uremia-related ones (chronic inflammation, oxidative stress, endothelial dysfunction, anemia, fluid overload, and uremia toxins) is found. Many studies have shown increased morbidity and mortality from cardiovascular causes in adults with CKD. In children, because hard endpoints, such as stroke or cardiovascular death, are rarely evaluated, surrogate endpoints, such as left ventricular hypertrophy (LVH), are more frequently observed [7].

The aim of the study was to assess the association between oxidative stress biomarkers and cardiovascular risk factors and left ventricular hypertrophy in children with chronic kidney disease.

## 2. Material and Methods

Medical examinations were carried out from June 2008 to February 2011. The study was performed in accordance with the Declaration of Helsinki of 1975 for Human Research and approved by the Bioethical Committee of the Jagiellonian University (KBET/17/B/2006). The parents and patients were informed about the objective and method of performing the study and gave their informed consent.

### 2.1. Subjects

The inclusion criterion was the age of 0–21 years and diagnosed chronic kidney disease. The exclusion criteria were lack of consent of the patient or parents, congenital heart defects or other primary heart diseases, acute infections, or failure of other organs.

### 2.2. Blood Sampling and Biochemical Analysis

On admission blood samples were taken from all patients (fasting for 12 hours). Three samples were collected and centrifuged and plasma and serum were frozen at −80°C. Biochemical analyses necessary to determine kidney function were performed and urea, creatinine, cystatin C, electrolytes, albumin, aldosterone, and lipids concentrations were measured; plasma renin activity (PRA) was assessed. On the basis of serum creatinine and cystatin C, an estimated glomerular filtration rate (eGFR) with the Schwartz [8] and Filler [9] formulas was calculated. Patients were divided into groups depending on the stage of CKD [group 1: CKD stages 1 + 2 (GFR > 60), group 2: CKD stage 3 (GFR = 30–59), group 3: CKD stage 4 (GFR = 15–29 mL/min/1.73 m^2^), group 4: dialyzed children].

To assess oxidative stress the concentration of oxidized LDL particles, as an effect of lipid oxidation, and the concentration of carbonyl groups resulting from oxidation of proteins were used. Concentrations of serum high sensitive C-reactive protein (hsCRP) (R&D Systems, USA); oxLDL (Mercodia Inc., Sweden); protein carbonyl groups (Cayman Chemical Company, USA); and intercellular adhesion molecule-1 (ICAM-1) (R&D Systems, USA) were determined with enzyme-linked immunosorbent assay (ELISA). Insulin levels (BioSource, Belgium) were measured using the IRMA method.

### 2.3. Anthropometric and Blood Pressure Measurements

During each visit, anthropometric parameters of patients, weight, height, and waist circumference, were measured. BMI was calculated by dividing weight in kilograms by height in meters squared. 24 h blood pressure monitoring (ambulatory blood pressure measurement, ABPM) using SpaceLabs 90207 device and cuff of appropriate size was performed. Blood pressure measurements were taken in an interval of 20 minutes during the day and every 30 minutes during the night. With the help of a licensed ABPM program mean values of systolic (SBP), diastolic (DBP), and mean blood pressure (mean arterial pressure, MAP) for the whole day were calculated. Hypertension was defined as BP values equal to or exceeding the 95th percentile for gender, age, and height [10]. The absolute values of height, weight, and BMI measurements were converted to *z*-scores based on data published by Palczewska and Niedźwiecka [11].

### 2.4. Left Ventricular Hypertrophy Assessment

Echocardiographic examinations were performed by an experienced cardiologist using HP 5500 unit with S4 and S8 variable frequency probes. In children on chronic hemodialysis echocardiography was performed on the day between two hemodialysis procedures, while in children on peritoneal dialysis, it was performed during the daily exchange, with a low volume of dialysate in the peritoneal cavity.

Left ventricular end-diastolic dimension (LVEDd), interventricular septal thickness at end diastole (IVSd), and left ventricular posterior wall thickness at end diastole (LVPWd) were measured by 2-dimensional guided M-mode echocardiography using the parasternal short-axis view at the level of the papillary muscles. Diameters and thickness were corrected for body surface area (BSA) and normal ranges were assessed according to values published by Kampmann et al. [12].

LV mass (LVM) was calculated by the formula described by Devereux and Reichek [13]. LVM *z*-score for height was calculated according to the method described by Foster et al. [14]. LVM index (LVMI) was obtained by dividing LVM by height^2.7^ to normalize and linearize the relationship between LVM and height [15]. LV hypertrophy (LVH) was diagnosed when LVMI was over the 95th percentile for healthy children [16]. We used age-specific cut-off values provided by Khoury et al. [17].

### 2.5. Statistical Analysis

Qualitative values were compared by the chi-square test. Because data of the majority of variables did not show normal distribution, they are presented as median [25th–75th percentile]. Differences between the groups were compared using the Kruskal-Wallis test. Spearman's rank correlation was used to relate levels of kidney function and oxidative stress markers. Statistical calculations were performed using a commercially available statistical package (Statistica PL). A value of *p* < 0.05 was considered significant in all statistical analyses.

## 3. Results

The studied group consisted of 65 patients (41 boys and 24 girls) aged 1.4 to 18.6 (mean 11.2) years with stage 1 to stage 5 CKD, who were under constant medical control in the University Children's Hospital in Krakow. Among diseases leading to the development of CKD in the examined children, the highest prevalence was noted in congenital abnormalities of the kidney and urinary tract, 31 (47.7%), followed by glomerulonephritis, 8 (12.3%), cystic disease, 7 (10.8%), and others, 19 (29.2%).

Clinical data and basic kidney function parameters depending on the stage of chronic kidney disease are presented in Table 1.

Several parameters of possible mechanisms (PRA, aldosterone, endothelial dysfunction-ICAM-1, inflammation-hsCRP, and hyperinsulinism) associated with oxidative stress were analyzed according to CKD stage in the studied group. There was a significant difference in median oxLDL concentration between CKD stages 3 and 5 (75.81 versus 98.89 U/L; *p* = 0.019). There were no significant differences in the concentration of most evaluated parameters (Table 2).

In the studied group 41 out of 65 patients were treated with angiotensin-converting enzyme inhibitors (ACEI) or angiotensin receptor blocker (ARB), 63.6% in stages 1 + 2, 72.2% in stage 3, 64.3% in stage 4, and 54.5% in stage 5, respectively. There were no differences between children on ACEI or ARB and without this treatment (data on request).

The average concentration of oxidized LDL was 86.94 U/L and of carbonyl groups 1.69 nmol/mg. Elevated concentrations of protein carbonyl groups (>4 nmol/mg) were observed in 6 of 54 patients. The median oxLDL concentration was significantly higher in girls than in boys (99.86 versus 81.06 U/L; *p* = 0.024).

In the studied population there was no correlation between oxLDL and age, CKD duration, weight, body mass index (BMI) in *z*-score, urea, high-density lipoprotein (HDL), albumin, insulin, hsCRP and ICAM-1, and aldosterone concentrations. Correlations of investigated parameters (aldosterone, PRA, carbonyl group, oxLDL, ICAM-1, hsCRP, and insulin) with kidney function markers were performed. The most pronounced correlations were found for oxLDL: its concentration significantly correlated with creatinine (*R* = 0.246; *p* = 0.048), cystatin (*R* = 0.346; *p* = 0.006), and eGFR calculated on their basis (*R* = −0.266; *p* = 0.032 and *R* = −0.296; *p* = 0.027, resp.). Furthermore oxLDL strongly correlated with total cholesterol (*R* = 0.500; *p* < 0.001), TGL (*R* = 0.524; *p* < 0.001), LDL (*R* = 0.446; *p* < 0.001), and 24 hour blood pressure values of SBP (*R* = 0.492; *p* = 0.002), DBP (*R* = 0.515; *p* < 0.001), and MAP (*R* = 0.537; *p* < 0.001) and negatively with PRA (*R* = −0.264; *p* = 0.038). hsCRP correlated significantly with creatinine (*R* = 0.266; *p* = 0.033), while insulin correlated with creatinine (*R* = 0.333; *p* = 0.009) and cystatin C (*R* = 0.337; *p* = 0.010) concentrations and eGFR calculated with the Filler formula (*R* = −0.422;  *p* = 0.002).

Patients were divided into 4 groups depending on the quartiles of oxLDL concentration and clinical, biochemical, and echocardiographic parameters between groups were compared. Children with high oxLDL concentrations were characterized by significantly higher blood pressure, triglycerides, and total and LDL cholesterol levels. In this group also higher left ventricular mass was found (Table 3).

Echocardiographic examinations revealed LVH in 34 children. There were no significant differences in median carbonyl groups concentrations between children with and without LVH (1.17 (0.62; 1.89) versus 1.29 (0.85; 1.96) nmol/mg; *p* = 0.567). A trend toward higher median oxLDL values in children with LVH was present (88.60 (76.24; 107.07) versus 81.06 (61.13; 98.35) U/L; *p* = 0.084), although it did not reach statistical significance.

**Figure 1:**
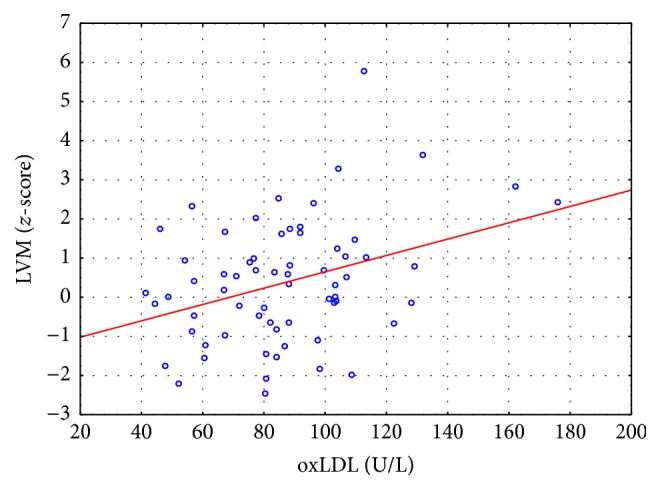
Scatterplot presenting the correlation between oxLDL concentrations and left ventricular mass (LVM) (*z*-score).

There was a significant correlation between oxLDL concentration and LVPWT (*z*-score) (*R* = 0.258; *p* = 0.038). In the univariate analysis, LVM in *z*-scores correlated significantly with eGFR (*R* = −0.427; *p* = 0.001), MAP (*z*-score) (*R* = 0.487; *p* = 0.002), albumin (*R* = −0.363; *p* = 0.004), and oxLDL concentrations (*R* = 0.299; *p* = 0.016) (Figure 1). In the multivariate analysis, the single independent parameter was MAP (*B* = 0.383; *p* = 0.001). After exclusion of MAP from the equation 3 parameters correlated independently with LVM *z*-score (eGFR: *B* = −0.01; *p* = 0.034; albumin: *B* = −0.025; *p* = 0.047; and oxLDL: *B* = 0.023; *p* = 0.001).

## 4. Discussion

This is, to our knowledge, the first study comparing oxidative stress markers and left ventricular hypertrophy in children with chronic kidney disease.

Despite the enormous technological progress and the introduction of new medications into the treatment of patients with impaired renal function, this group is characterized by an increased cardiovascular risk. Mortality associated with cardiovascular causes is higher in children and adults with CKD compared to the general population [18]. According to data from the USA, the estimated survival time of children on dialysis is 40–60 years shorter than healthy peers [19].

CKD patients are exposed to high prevalence of traditional cardiovascular risk factors as well as nontraditional ones, such as inflammation, oxidative stress, and endothelial dysfunction. These factors are responsible for accelerated atherosclerosis and heart damage. Fruchart et al. proposed a division of atherosclerosis risk factors into the old, the old/new, and the new [20]. The authors included into new agents, among others, triglycerides, oxidized LDL and anti-oxidized LDL antibodies, lipoprotein (a), homocysteine, and hsCRP, which indicates the role of oxidative stress and inflammation in atherosclerosis. Numerous studies [21–24] have shown increased concentrations of oxidative stress markers, such as advanced products of protein oxidation, malondialdehyde, and isoprostanes in patients with chronic kidney disease. Children on dialysis have demonstrated reduced antioxidant enzymes activity and decreased levels of trace elements [25]. According to different authors, oxidative stress plays a central role in the development and accelerated progression of atherosclerosis in patients with impaired renal function [23].

Our data suggest that uremia per se is a significant contributor to oxidative stress. In the studied group of children with chronic kidney disease a significant correlation between the concentrations of oxidized LDL and serum creatinine and cystatin C was demonstrated. The highest median concentration of oxLDLs was found in the group of children undergoing dialysis. Furthermore, a significant influence of traditional cardiovascular risk factors, hypertension and lipid disturbances, on the severity of oxidative stress in children with CKD was shown. oxLDL concentration correlated strongly with 24 hour systolic, diastolic, and mean arterial pressure values and with total cholesterol, triglycerides, and LDL cholesterol levels. The lack of correlation between kidney function impairment and aldosterone level as well as plasma renin activity is probably connected with wide use of nephroprotection, starting at the early stage of chronic kidney disease. From the studied population 63% of children were treated with ACEI or ARB.

Statins are becoming more widely used in children, especially in those with familial hypercholesterolemia [26]. Given the high cardiovascular risk in patients with CKD and lack of efficacy of dietary restrictions in lipid normalization, it seems reasonable to execute clinical trials and determine the indications for statin therapy in these children and adolescents. A few recent studies have demonstrated that the use of statins may not only inhibit cholesterol synthesis but also have important pleiotropic effects, such as antioxidant and cytoprotective abilities. In the study of Chang et al. a significant reduction in CRP levels after 8 weeks of simvastatin therapy was observed in hemodialysis patients, which reflects the anti-inflammatory effect of statins [27]. In the study by Kumar et al., in a retrospective analysis of 257 dialyzed patients, the relation between statin therapy and lower CRP levels was found [28]. Furthermore, in another study a significant relationship between statin use and reduction of IL-6 levels was identified [29]. The result of anti-inflammatory effect of statins treatment may exceed beyond their lipid lowering effect in patients with CKD, but there is insufficient data for the pediatric population.

Over the last decades, several research studies investigating the role of oxidative stress in chronic kidney disease in adults were undertaken [30]. Müller et al. examining DNA damage showed a significantly higher degree of oxidative stress in hemodialyzed patients, compared to healthy volunteers [31]. Kaneda et al. found elevated AOPP concentrations in patients with ischemic heart disease and in those treated with hemodialysis. It should be pointed out that the severity of coronary heart disease correlated with AOPP quartiles [32]. In hemodialyzed patients the concentration of malondialdehyde (MDA)—a measure of lipid peroxidation—was significantly higher than that in patients with CKD treated conservatively and healthy subjects [33]. On the other hand, the concentration of an antioxidant—superoxide dismutase—was reduced. The combination of increased oxidative stress and lipid disorders leads to the progression of the process of atherosclerosis in patients with chronic kidney disease. Sakata and colleagues found an increased accumulation of advanced glycation end products in atherosclerotic lesions—from intimal thickening to atherosclerotic plaque—in the aorta of people with end-stage renal disease [34]. In the postmortem examination of aorta sections an increased content of pentosidine and MDA in the fraction of elastin in patients on hemodialysis was detected. The modification of elastin in the processes of glycoxidation and lipid peroxidation could lead to vascular lesions exaggeration in patients with end-stage renal disease [35].

The main factor influencing the increase in left ventricular mass in our study was elevated blood pressure. Several other parameters such as high oxLDL, low albumin concentrations, and low eGFR also correlated with LVM. The studied group of children with high oxLDL levels had significantly higher LVM and left ventricular posterior wall thickness. Future studies are needed to evaluate oxLDL as a biomarker of oxidative stress target organ damage in children. Holvoet et al. proposed evaluating the concentration of circulating oxidized LDLs as means of a more accurate cardiovascular risk assessment. Adults with coronary artery disease confirmed in angiography had significantly higher concentrations of oxLDLs. A significant correlation between oxLDLs and most of Framingham risk factors was also demonstrated [36]. In hemodialyzed patients, particularly vulnerable to oxidative stress, a beneficial effect of antioxidant usage in the form of large vitamin E doses on secondary prevention was shown in the SPACE study [37]. Treatment with vitamin E for a period of about two years reduced the risk of myocardial infarction, ischemic stroke, and peripheral vascular disease. The supply of a different antioxidant (N-acetyl-cysteine) led to a reduction in intracellular oxidative stress and the incidence of apoptosis in T cells in children on chronic hemodialysis [38].

In the studied population a significant correlation between hsCRP and serum creatinine concentrations was found. It should be stressed out that in children recorded hsCRP concentrations did not exceed 3 mg/L, a value that indicates an increased cardiovascular risk associated with the severity of chronic inflammation. In adults on hemodialysis significantly higher concentration of carbonyl groups in comparison to healthy subjects was observed. Danielski and coauthors suggested that increased oxidative stress associated with inflammation and phagocytic cell activation might preferentially increase aldehyde formation and oxidize thiol groups in proteins rather than promote lipid peroxidation [39]. In adults with diabetes mellitus protein carbonyl (PCO) content was higher than that in healthy controls and the hemodialysis procedure caused additional elevation of PCO levels [40]. The lack of a significant increase in protein carbonyl content in children with advanced CKD compared to adults can result from various diseases leading to impaired renal function. In adults, the most common causes of CKD are diabetes mellitus and long-lasting hypertension and in children, congenital abnormalities of kidney and urinary tract. In our study there was no correlation between insulin and oxLDL concentrations.

Chronic kidney disease is frequently accompanied by decreased albumin levels, both in adults and in children [41, 42]. Serum albumin concentration was found to be an independent predictor of mortality risk in a broad range of clinical and research settings in adults [43], especially in those with end-stage renal disease [44]. This increase in mortality was independent of malnutrition, a condition that until recently was thought to be the reason for reduced albumin levels [44]. Albumin is postulated to be major and predominant circulating antioxidant [45]. Several researchers have evaluated the relation between hypoalbuminemia and oxidative stress in adults with CKD; however no data is available for the pediatric population. Levels of inflammatory and oxidative stress biomarkers were increased in hypoalbuminemic compared with normoalbuminemic end-stage renal disease adults undergoing hemodialysis in a study by Danielski et al. [39]. Kaneko and coauthors found significantly lower serum levels of biological antioxidant potentials in adults with idiopathic nephrotic syndrome [46]. In our study we found no correlation between albumin and oxLDL concentrations. Low albumin is related to overhydration and, as a result, high blood pressure [42] and left ventricular hypertrophy [47]. We demonstrated a significant negative correlation between albumin concentration and left ventricular mass expressed in *z*-score.

In the early stages of the atherosclerotic process there is an activation of adhesion molecules, which promotes adhesion of monocytes to the vascular wall and their migration to the intima. As a result of chronic inflammation adhesion molecules belonging to the immunoglobulin family: ICAM-1 (intercellular adhesion molecule-1) and VCAM-1 (vascular adhesion molecule-1) are being expressed on endothelial cells. In the work of Ridker et al. the growing concentration of soluble ICAM-1 adhesion molecule was one of the major cardiovascular risk factors in postmenopausal women and the risk increased 2.6-fold between the lowest and highest quartiles of ICAM-1 levels [48]. In the studied population of children with CKD, no influence of the degree of renal function impairment on plasma ICAM-1 was found. Furthermore, no correlation of ICAM-1 with oxidative stress biomarkers was shown.

## 5. Conclusions

In children with chronic kidney disease an increase in the concentration of oxidized LDLs with the progression of the disease was found. This biomarker of oxidative stress was strongly correlated with 24 hour blood pressure values, triglycerides, and total and LDL cholesterol levels. Oxidized LDLs seem to be valuable markers of oxidative stress in CKD patients, correlating with left ventricular hypertrophy. In contrast to adults, the protein carbonyl content did not increase in advanced stages of CKD.

## Supplementary Material

In the supplementary materials 4 groups of primary kidney diseases leading to renal impairment in the studied group of children (congenital abnormalities of the kidney and urinary tract (CAKUT), glomerulonephritis, cystic disease and others) are presented.

## Figures and Tables

**Table 1:** Basic clinical data and kidney function parameters depending on CKD stage in the investigated group of 65 patients.

Parameter	CKD stage				*p* value
	1 + 2 (*n* = 11)	3 (*n* = 18)	4 (*n* = 14)	5 (*n* = 22)	
Age (years)	10.51 (5.04; 16.08)	11.33 (5.15; 16.33)	12.01 (8.70; 15.99)	11.61 (8.51; 15.20)	0.820

Height (*z*-score)^*∗*^	0.078 (−0.300; 0.569)	−0.716 (−2.303; 0.060)	−0.868 (−1.484; −0.210)	−1.495 (−3.444; −0.130)	**0.013**

Body mass (*z*-score)^*∗∗*^	−0.015 (−0.409; 0.419)	−0.980 (−1.635; 0.009)	−0.464 (−1.054; 0.228)	−1.587 (−3.714; −0.828)	**0.002**

BMI (kg/m^2^)	16.5 (15.8; 22.1)	16.6 (14.7; 19.5)	18.1 (16.0; 20.4)	15.9 (14.5; 17.2)	0.224

BMI (*z*-score)^*∗∗∗*^	0.316 (−0.318; 0.712)	−0.779 (−1.636; 0.433)	0.079 (−0.638; 0.712)	−1.252 (−1.599; −0.621)	**0.020**

Creatinine (*µ*mol/L)^*∗∗∗∗*^	69.3 (34.0; 93.8)	113.8 (95.0; 146.4)	274.9 (203.8; 311.9)	501.8 (414.3; 869.0)	**<0.001**

Cystatin C (mg/L)^*∗∗∗∗*^	0.86 (0.68; 1.23)	1.45 (1.17; 1.86)	2.6 (2.23; 3.01)	4.81 (4.14; 6.53)	**<0.001**

eGFR Filler^*∗∗∗∗*^(mL/min/1.73 m^2^)	109.46 (72.62; 141.28)	60.13 (45.64; 76.81)	29.49 (26.19; 36.49)	14.77 (9.78; 18.43)	**<0.001**

Values presented as median (25th–75th percentile).

BMI—body mass index; eGFR—estimated glomerular filtration rate.

^*∗*^Statistically significant differences between stages 1 + 2 and 4, 1 + 2 and 5.

^*∗∗*^Statistically significant differences between stages 1 + 2 and 3, 1 + 2 and 5, 4 and 5.

^*∗∗∗*^Statistically significant differences between stages 4 and 5.

^*∗∗∗∗*^Statistically significant differences between all stages.

**Table 2:** Selected parameters depending on CKD stage in the investigated group of 65 patients.

Parameter	CKD stage				*p* value
	1 + 2 (*n* = 11)	3 (*n* = 18)	4 (*n* = 14)	5 (*n* = 22)	
hsCRP (ng/mL)	171.6 (128.7; 464.3)	252.4 (106.5; 2574.9)	338.9 (143.0; 771.6)	365.6 (187.2; 878.1)	0.397

Carbonyl groups (nmol/mg)	1.15 (0.54; 1.32)	1.24 (0.87; 1.69)	1.64 (0.73; 2.41)	1.23 (0.66; 2.05)	0.454

oxLDL (U/L)^*∗*^	80.65 (60.56; 109.68)	75.81 (56.66; 97.87)	82.31 (75.48; 91.92)	98.89 (82.16; 108.73)	**0.030**

Aldosterone (pg/mL)	186.1 (103.5; 298.7)	272.8 (142.3; 662.1)	471.00 (169.0; 1073.1)	192.3 (86.4; 711.4)	0.250

PRA (ng/mL/h)	4.99 (1.25; 8.38)	6.88 (3.41; 12.51)	4.69 (1.64; 9.98)	3.88 (2.01; 7.94)	0.521

ICAM-1 (ng/mL)	305.7 (289.4; 354.3)	319.1 (291.8; 446.3)	322.1 (290.3; 337.7)	329.2 (267.3; 408.9)	0.922

Insulin (*μ*IU/mL)	9.0 (7.0; 10.6)	9.5 (8.1; 15.7)	12.8 (10.1; 16.8)	13.1 (6.9; 16.3)	0.162

Albumin (g/L)	45.0 (41.0; 47.0)	45.50 (41.0; 47.5)	46.8 (45.0; 49.1)	44.1 (37.7; 47.1)	0.127

Values presented as median (25th–75th percentile)

hsCRP—high sensitive C-reactive protein; oxLDL—oxidized low-density lipoprotein; PRA—plasma renin activity; ICAM-1—intercellular adhesion molecule-1.

^**∗**^Statistically significant differences between stages 3 and 5.

**Table 3:** Investigated clinical, biochemical, and echocardiographic parameters in the groups with oxLDL quartiles.

Parameter	oxLDL (U/L)				*p* value
	41.4–67.4 (*n* = 16)	67.5–84.8 (*n* = 17)	86.0–103.5 (*n* = 16)	103.6–176.1 (*n* = 16)	
SBP 24 h (*z*-score)	0.11 (−2.64; 0.53)	−0.62 (−0.92; 0.56)	−0.33 (−1.41; 1.30)	1.86 (1.03; 2.73)	**0.009**

DBP 24 h (*z*-score)	−0.06 (−1.80; 0.27)	−0.77 (−2.14; 1.37)	−0.46 (−1.40; 1.78)	2.42 (1.16; 4.64)	**0.004**

MAP 24 h (*z*-score)	0.42 (−1.82; 0.79)	−0.32 (−1.09; 1.04)	0.00 (−0.86; 1.76)	2.32 (2.01; 4.45)	**0.003**

Total chol. (mmol/L)	4.19 (3.63; 4.81)	4.48 (4.13; 5.01)	4.67 (4.08; 5.62)	6.19 (5.39; 7.50)	**0.001**

TGL (mmol/L)	1.21 (0.90; 1.62)	1.30 (1.08; 1.47)	1.69 (1.28;2.35)	2.62 (1.63; 3.70)	**0.001**

HDL (mmol/L)	1.35 (1.08; 1.76)	1.35 (1.17; 1.76)	1.19 (1.03;1.43)	1.26 (0.97; 1.62)	0.417

LDL (mmol/L)	2.07 (1.70; 2.59)	2.43 (1.99; 2.64)	2.49 (2.08; 3.39)	3.26 (2.57; 4.01)	**0.005**

Cystatin (mg/L)	1.36 (1.09; 2.97)	2.36 (1.17; 3.46)	2.55 (1.61; 4.07)	4.24 (1.48; 6.12)	0.053

Albumin (g/L)	46.0 (44.1; 48.0)	46.0 (44.0; 47.7)	45.0 (38.3; 47.0)	43.0 (34.0; 47.6)	0.320

hsCRP (ng/mL)	267.5 (159.5; 688.2)	321.9 (162.5; 553.4)	459.2 (104.3; 1270.2)	273.2 (121.5; 486.4)	0.939

ICAM-1 (ng/mL)	295.5 (267.3; 339.4)	303.0 (288.5; 346.4)	339.9 (316.0; 405.8)	326.4 (278.4; 396.7)	0.373

Carbonyl groups (nmol/mg)	1.39 (0.87; 1.69)	1.73 (0.50; 2.25)	1.23 (0.96; 1.87)	0.92 (−0.54; 1.75)	0.736

LVM (*z*-score)	0.05 (−1.06; 0.76)	−0.27 (−0.99; 0.64)	0.73 (−0.41; 1.70)	1.01 (−0.05; 2.61)	**0.038**

LVEDd *z*-score	0.17 (−0.61; 0.85)	0.19 (−0.19; 0.61)	−0.03 (−0.60; 0.57)	0.22 (−0.31; 0.83)	0.765

IVSd *z*-score	0.99 (.07; 1.42)	0.31 (−0.06; 0.93)	1.06 (0.18; 1.72)	1.08 (0.56; 2.07)	0.082

LVPWT *z*-score	0.75 (0.18; 1.40)	1.32 (0.26; 1.58)	1.40 (0.50; 2.19)	1.45 (0.65; 2.37)	0.281

Values presented as median (25th–75th percentile).

SBP 24 h—systolic blood pressure 24 h; DBP 24 h—diastolic blood pressure 24 h; MAP 24 h—mean arterial pressure 24 h; TGL—triglycerides; HDL—high-density lipoprotein; LDL—low-density lipoprotein; hsCRP—high sensitive C-reactive protein; oxLDL—oxidized low-density lipoprotein; ICAM-1—intercellular adhesion molecule-1; LVM—left ventricular mass; LVEDd—left ventricular end-diastolic dimension; IVSd—interventricular septum at end diastole; LVPWd— left ventricular posterior wall thickness at end diastole.
