# Supplementary material for: Oxidative Stress Biomarkers and Left Ventricular Hypertrophy in Children with Chronic Kidney Disease
Source: Oxid Med Cell Longev. 2016 Jan 18;2016:7520231. doi: 10.1155/2016/7520231 (PMC4739446; doi:10.1155/2016/7520231)
Supplement: Supplementary file 1 — In the supplementary materials 4 groups of primary kidney diseases leading to renal impairment in the studied group of children (congenital abnormalities of the kidney and urinary tract (CAKUT), glomerulonephritis, cystic disease and others) are presented. [file 7520231.f1.docx]

Suppl. Table 1. Diseases leading to the development of CKD in the examined children.

| **CAKUT** **(n=31)** | **Glomerulonephritis** **(n=8)** | **Cystic disease** **(n=7)** | **Others** **(n=19)** |
| --- | --- | --- | --- |
| - posterior urethral valve (n=13) - renal hypo/dysplasia (n=7) - single kidney agenesis (n=2) - reflux (n=6) - obstructive nephropathy (n=3) | - focal glomerulosclerosis (n=4) - mesangioproliferative glomerulonephritis (GN) (n=1) - progressive GN (n=1) - lupus GN (n=2) | - cystic degeneration of the kidney (n=7) | - post-chemotherapy damage (n=4) - hemolytic-uremic syndrome (n=3)  - nephronophtisis (n=2) - vasculitis (n=1)  - nail-patella syndrome (n=1) - neuroblastoma with unilateral nephrectomy (n=1) - primary hypoparathyroidism (n=1) - pyelonephritis (n=1)  - bladder exstrophy (n=1) - prune belly syndrome (n=1) - Fanconi syndrome (n=1) - Lowe syndrome (n=1) - interstitial nephritis (n=1) |

CKD - chronic kidney disease; CAKUT - congenital anomalies of the kidney and urinary tract
